# Na^+^ riboswitches regulate genes for diverse physiological processes in bacteria

**DOI:** 10.1038/s41589-022-01086-4

**Published:** 2022-07-25

**Authors:** Neil White, Harini Sadeeshkumar, Anna Sun, Narasimhan Sudarsan, Ronald R. Breaker

**Affiliations:** 1 Department of Molecular, Cellular and Developmental Biology, Yale University, New Haven, CT USA; 2 Howard Hughes Medical Institute, Yale University, New Haven, CT USA; 3 Department of Molecular Biophysics and Biochemistry, Yale University, New Haven, CT USA

**Keywords:** Metals, Bacteria, Riboswitches, RNA

## Abstract

Organisms presumably have mechanisms to monitor and physiologically adapt to changes in cellular Na^+^ concentrations. Only a single bacterial protein has previously been demonstrated to selectively sense Na^+^ and regulate gene expression. Here we report a riboswitch class, previously called the ‘DUF1646 motif’, whose members selectively sense Na^+^ and regulate the expression of genes relevant to sodium biology. Many proteins encoded by Na^+^-riboswitch-regulated genes are annotated as metal ion transporters, whereas others are involved in mitigating osmotic stress or harnessing Na^+^ gradients for ATP production. Na^+^ riboswitches exhibit dissociation constants in the low mM range, and strongly reject all other alkali and alkaline earth ions. Likewise, only Na^+^ triggers riboswitch-mediated transcription and gene expression changes. These findings reveal that some bacteria use Na^+^ riboswitches to monitor, adjust and exploit Na^+^ concentrations and gradients, and in some instances collaborate with c-di-AMP riboswitches to coordinate gene expression during osmotic stress.

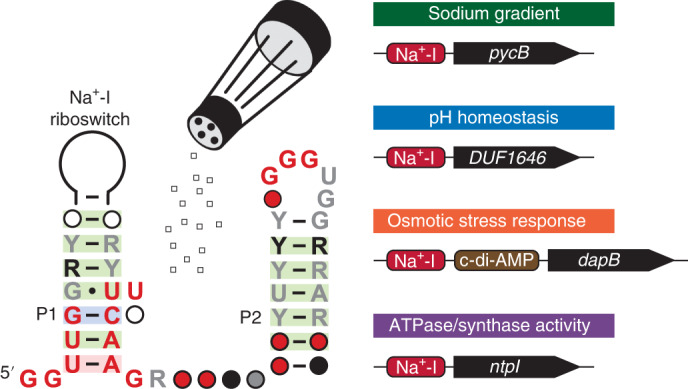

## Abstract (web summary)

Only one protein factor is known that senses Na^+^ and controls gene expression. The Breaker Laboratory describes a bacterial riboswitch class selective for Na^+^ that regulates genes important for Na^+^ homeostasis, pH maintenance, osmotic stress response and ATP synthesis.

## Main

Over 50 distinct classes of riboswitches^1,2^ have been discovered in bacteria that selectively sense their target ligands and regulate gene expression. Each class is defined by its structural and functional characteristics, wherein highly conserved nucleotide sequences and substructures of its aptamer domain are used to form a specific binding pocket for its target ligand. The most common riboswitch classes predominantly sense nucleotide-derived enzyme cofactors or other common nucleotide-like metabolites or signaling molecules^2^. These findings strongly indicate that the most abundant and widely distributed riboswitch classes might have emerged during the RNA World^3^, which is proposed to have been a time early in life’s history that was dominated by RNA-guided metabolism^4^.

Based on the abundances of known riboswitch classes, it is estimated that many thousands of additional classes remain to be discovered^1,3^, although most of these are likely to be exceedingly rare. If true, this leaves open the possibility that new riboswitch classes might exist that sense many additional fundamental metabolites, other biologically relevant molecules, and elemental ions. Specifically, the opportunity for the discovery of ion-sensing riboswitches appears to be substantial, given that many Earth-abundant monoatomic cations and anions are either productively exploited by living systems or can increase in concentration in certain environments to become toxic.

Numerous classes of ion-sensing riboswitches already have been reported, including those that bind to Mg^2+^ (refs. ^5,6^), Mn^2+^ (refs. ^7,8^), Ni^2+^ and Co^2+^ (ref. ^9^), Fe^2+^ (ref. ^10^) and F^−^ (ref. ^11^). Notably absent from this growing collection of ion-responsive riboswitches are examples that sense alkali metal cations such as Na^+^ and K^+^, both of which are present in cells from all domains of life. Indeed, bacterial cells must adjust the concentration of Na^+^ in cells for various reasons^12–14^. For example, Na^+^ is maintained at a low concentration relative to its typical concentration outside cells^15^. Na^+^ also participates in the operation of proton pumps that help maintain the pH of cells^13,16^. Furthermore, in some bacterial species, a Na^+^ gradient can be harnessed to synthesize ATP^17^.

There are very few descriptions of protein factors that both selectively sense Na^+^ and regulate gene expression. The only experimentally validated Na^+^-selective gene control factor is the NhaR protein from *Escherichia coli*^18^, although other proteins have been proposed to serve similar functions^19^. The dearth of reported Na^+^-selective protein genetic factors in bacteria could be simply due to limited exploration of such regulatory systems. However, it is also prudent to consider the possibility that some bacteria instead use Na^+^-dependent riboswitches to regulate genes related to Na^+^ homeostasis.

**Fig. 1:**
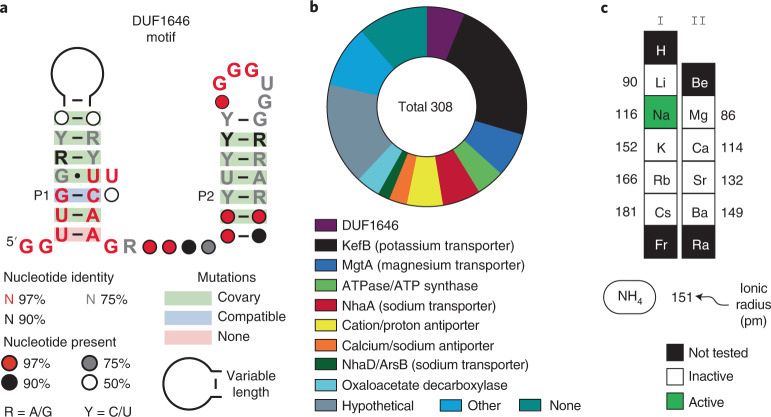
The DUF1646 motif associates with genes related to metal cation transport. **a**, Consensus sequence and secondary structure model of the riboswitch candidate class called the DUF1646 motif. The consensus model is based on the alignment of 308 nonredundant examples. **b**, Existing annotations of the protein products whose genes are associated with DUF1646 motif RNAs. **c**, Alkali (I) and alkaline earth (II) cations considered as candidate ligands for the putative aptamer formed by DUF1646 motif RNAs. Numbers represent the ionic radius (pm) in crystal form^50^ for the mono- or divalent forms of the elements indicated.

Through our ongoing efforts to identify additional riboswitch classes, we discovered the DUF1646 motif (Fig. 1a) among several phyla including Firmicutes, Proteobacteria, Acidobacteria and Verrucomicrobia^20^. Using comparative sequence analysis data, the RNA motif was predicted to form two extended base-paired substructures called P1 and P2. We speculated that two clusters of conserved nucleotides, located at the bottom of P1 and the loop of P2, might interact to form the ligand-binding aptamer domain for a riboswitch.

Although no natural Na^+^-selective RNA aptamers have been reported previously, nucleic acids that sense and respond to Na^+^ have been created by using directed evolution methods. For example, a class of deoxyribozymes (DNAzymes) has been isolated that cleaves an RNA phosphodiester linkage most efficiently in the presence of millimolar amounts of Na^+^ (ref. ^21^). Another collection of deoxyribozymes was reported with similar sequence, structure and functional characteristics that strongly select for Na^+^ (ref. ^22^). These findings indicate that nucleic acids have the structural sophistication needed to selectively bind Na^+^, even in the complex biochemical environment found inside cells. Consistent with this view is the fact that natural lysine riboswitch aptamers are known to selectively bind K^+^ as a coligand^23^.

An updated list of genes associated with DUF1646 motif RNAs (Fig. 1b) reveals that most are annotated as coding for transporters of alkali or alkaline earth ions. However, it is important to note that gene annotations in public databases are often inaccurate, particularly for those that code for transporters. For example, the sequences of the *kefB* genes commonly associated with the RNA motif are a better match for *napA* genes, which code for Na^+^/H^+^ antiporters^24^. Furthermore, some genes are annotated as oxaloacetate decarboxylase or as components of an ATP synthase apparatus, which are known to manipulate or exploit Na^+^ gradients^25,26^. These gene associations reinforce the originally proposed hypothesis^20^ that DUF1646 motif RNAs represent the first examples of Na^+^-responsive riboswitches.

Indeed, our biochemical and genetic findings described herein demonstrate that representatives of this riboswitch class bind Na^+^ and activate gene expression, but reject all other alkali and alkaline earth cations tested (Fig. 1c). These findings also reveal that some bacterial species make extensive use of RNA molecules to carry out the critical tasks of monitoring and adapting to changes in cellular Na^+^ concentrations. This advance opens new avenues for the study of factors and mechanisms relevant to Na^+^ homeostasis and exploitation.

## Results

### An orphan riboswitch candidate selectively senses Na^+^

The DUF1646 RNA motif represents a putative riboswitch class that was previously identified by using a computational pipeline to uncover new structured RNA domains in bacteria^20^. A single example (locus EF1492) of this orphan riboswitch candidate was also previously identified in *Enterococcus faecalis* by a transcriptomics analysis method called Term-seq^27^, which was implemented to identify regulatory RNA domains in bacteria of interest. We used a method called in-line probing^28,29^ (Methods) to assess whether Na^+^ binding selectively triggers structural changes in DUF1646 RNAs, which are characteristic of ligand-responsive riboswitches. Specifically, unstructured RNA regions undergo more rapid spontaneous RNA strand scission, and thus the pattern of RNA cleavage products visualized using PAGE can reveal structure-altering effects caused by ligand binding.

**Fig. 2:**
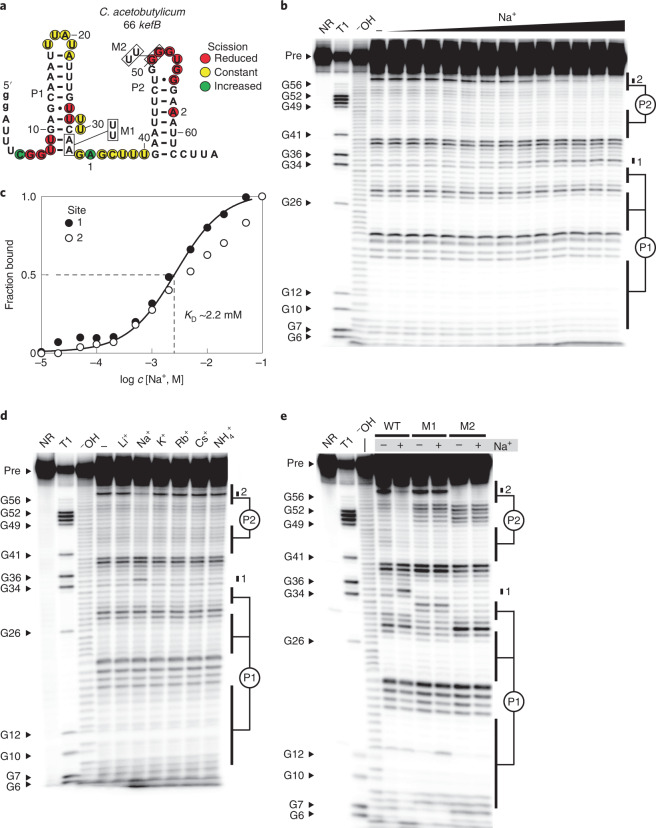
Selective binding of Na^+^ by a natural RNA aptamer. **a**, Sequence and secondary structure of the WT 66 *kefB* RNA construct used to assess ligand-binding characteristics of a DUF1646 motif RNA. The two lowercase letters designate guanosine nucleotides appended to the natural bacterial sequence from the *kefB* gene to facilitate efficient production by in vitro transcription. Mutant constructs M1 and M2 carry the boxed nucleotide changes at the sites indicated. Circles identify positions of notable strand scission from the in-line probing data depicted in **b**. **b**, PAGE separation of product bands resulting from in-line probing assay reactions reveal RNA shape changes induced by the addition of Na^+^. 5′ ^32^P-labelled precursor (Pre) RNA was subjected to no reaction (NR), partial digestion with RNase T1 (T1) (cleaves after G nucleotides), incubation in alkali conditions (^–^OH), or incubation in in-line probing reactions with buffer alone (including 2 mM MgCl_2_ but lacking KCl) (−) or supplemented with various amounts of Na^+^ ranging from 10 μM to 100 mM. Bands corresponding to some products generated by RNase T1 digestion are identified by the guanosine nucleotide position located immediately 5′ of the cleavage site. Bands of interest were mapped according to their sites of spontaneous strand scission on the RNA construct depicted in **a**. **c**, Plot of the fraction of RNA bound to Na^+^ as estimated from the in-line probing data depicted in **b**. Band intensities at sites 1 and 2 were used to generate the plot. The line depicts an idealized binding curve for a 1-to-1 interaction and a *K*_D_ of 2.2 mM. **d**, PAGE analysis of in-line probing assays using 5′ ^32^P-labelled 66 *kefB* RNA and conducted in the presence of various alkali metal cations or NH_4_^+^ at 10 mM. **e**, PAGE analysis of in-line probing assays using 5′ ^32^P-labelled WT 66 *kefB* RNA or the mutant M1 or M2 versions of this construct. In-line probing reactions were conducted in the absence (−) or presence (+) of 10 mM Na^+^.

A 5′ ^32^P-labelled RNA called 66 *kefB* (Fig. 2a) carrying a DUF1646 motif from the *kefB* gene of *Clostridium acetobutylicum* exhibits a pattern of spontaneous RNA cleavage (Fig. 2b) that matches the proposed secondary structure. Several product bands change in intensity as Na^+^ is added to a mixture containing 50 mM Tris-HCl (pH 8.3 at 20 °C) and 2 mM MgCl_2_. Most changes reveal a suppression of spontaneous cleavage in regions that exhibit the greatest RNA sequence conservation, such as nucleotides 6–9 (beginning of P1), nucleotides 27 and 28 (preceding the P1 bulge), nucleotides 50–55 (encompassing the conserved loop portion of P2) and nucleotide A58 in the right shoulder of P2. These Na^+^-responsive changes in RNA cleavage products demonstrate that Na^+^ structurally stabilizes the highly conserved core of the riboswitch aptamer.

In contrast, the banding pattern for the nucleotides joining the P1 and P2 stems indicates that this region is relatively unstructured regardless of Na^+^ supplementation. Indeed, the linkage following position A35 undergoes increased cleavage as Na^+^ concentration increases, suggesting this nucleotide becomes less structured. This pattern, along with the fact that the linker region lacks sequence and length conservation, suggests that the region joining P1 and P2 constitutes a passive, unstructured region that simply tethers the two main parts of the aptamer domain, wherein the aptamer portions in P1 and P2 form stable contacts only when the Na^+^ ligand is present.

**Extended Data Fig. 1:**
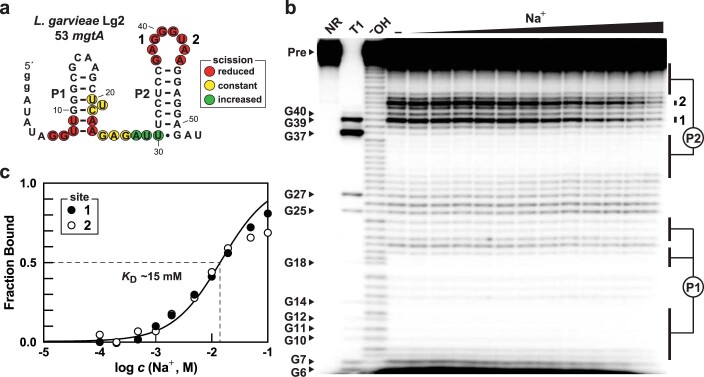
Na^+^-mediated structural modulation of a DUF1646 motif RNA representative from *Lactococcus garvieae* Lg2. **a**, Sequence and secondary structure model for the 53 *mgtA* construct derived from *L. garvieae* Lg2. Annotations are as described for Fig. 2a, with the in-line probing data derived from b. **b**, PAGE analysis of in-line probing reactions with trace amounts of 5′ ^32^P-labelled 53 *mgtA* RNA. Annotations are as described for Fig. 2b. **c**, Plot of the estimated fraction of RNA bound to Na^+^ generated by monitoring band intensity changes at sites 1 and 2 as depicted in b. The line depicts an idealized binding curve for a 1-to-1 interaction between RNA and Na^+^, yielding a *K*_D_ of ~15 mM. Additional annotations are as described for Fig. 2c.

To establish Na^+^ stoichiometry and apparent dissociation constant (*K*_D_) values, two bands that undergo the greatest change in intensity were quantified: position A35 (called site 1) and position A58 (site 2) (Fig. 2b). Band intensities were used to estimate the fraction of RNA bound to ligand, where fraction bound values of 0 and 1 were set to the intensities measured in the absence of added Na^+^ and the presence of 100 mM NaCl, respectively. This analysis yielded a curve that is most consistent with one-to-one binding between Na^+^ and 66 *kefB* RNA, with a *K*_D_ value of roughly 2.2 mM (Fig. 2c). Similar results were obtained for a homologous DUF1646 construct derived from another bacterial species *Lactococcus garvieae*, which exhibits a *K*_D_ value of around 15 mM (Extended Data Fig. 1).

**Extended Data Fig. 2:**
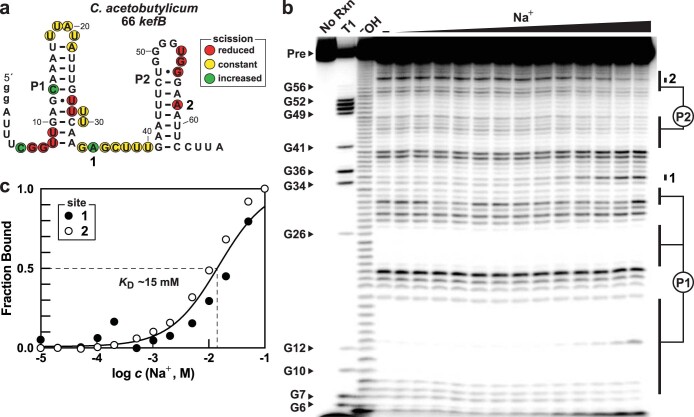
The 66 *kefB* RNA retains Na^+^ binding ability in the presence of 100 mM K^+^. **a**, Sequence and secondary structure model of the 66 *kefB* RNA construct with mapped in-line probing data generated in the presence of various Na^+^ concentrations. Data was derived from the assay results depicted in b. Additional annotations are as described for Fig. 2a. **b**, PAGE separation of product bands resulting from in-line probing assay reactions conducted using the 66 *kefB* RNA construct incubated with 0 to 100 mM NaCl. Additional annotations are as described for Fig. 2b except that 100 mM KCl was also present in each reaction. **c**, Plot of the fraction of RNA bound to Na^+^ as estimated from the in-line probing data depicted in b. Additional annotations are as described for Fig. 2c.

The 66 *kefB* RNA aptamer rejects all other alkali metal cations tested (Figs. 1c and 2d). For example, in-line probing assays typically contain 100 mM KCl to more closely simulate the ionic conditions present in cells^29^, but K^+^ was excluded in the assays described above to avoid possible interference with Na^+^ binding. However, a similar Na^+^-dependent response is observed for 66 *kefB* when in-line probing assays include 100 mM K^+^ (Extended Data Fig. 2), although the Na^+^ *K*_D_ is slightly poorer (roughly 15 mM). This demonstrates that the aptamer strongly discriminates against K^+^, which is by far the highest-concentration alkali cation naturally in cells and can reach concentrations above 100 mM (ref. ^30^). The RNA structure also rejects other alkali cations as well as ammonium (Fig. 2d), which is a small cation of similar ionic radius to Na^+^ and Li^+^ that could be present in cells under certain conditions.

**Extended Data Fig. 3:**
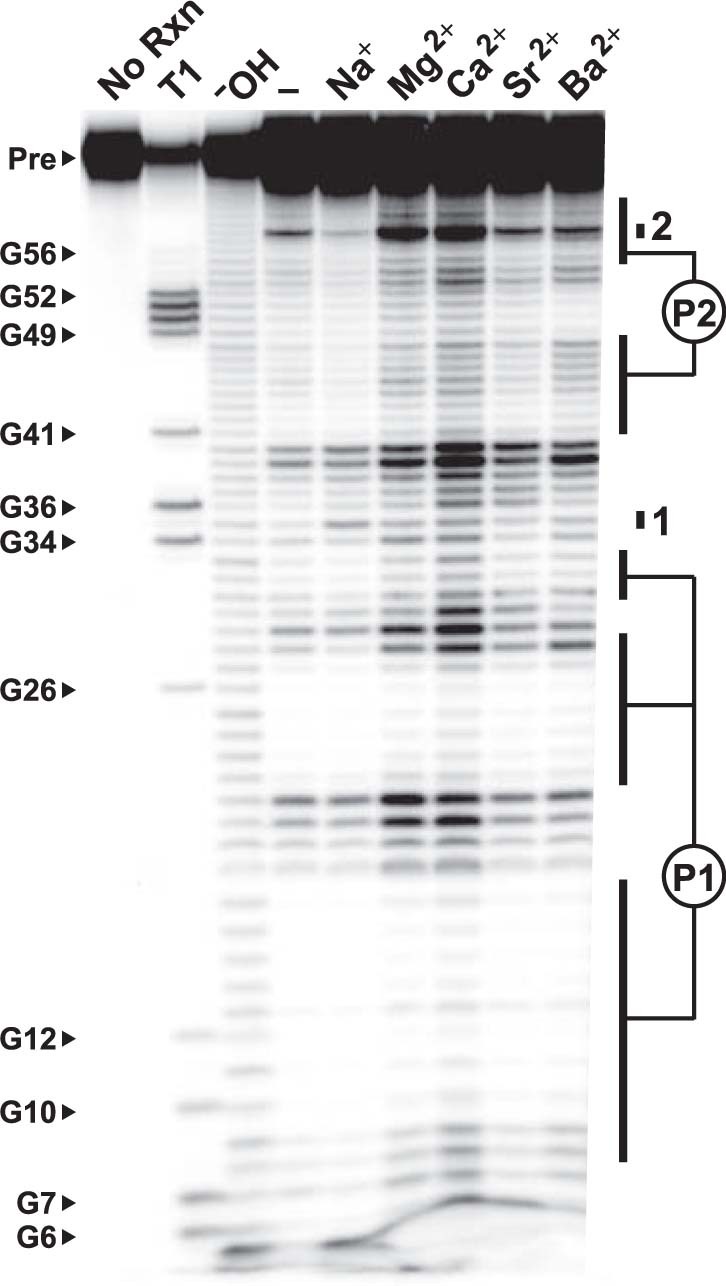
DUF1646 motif RNAs reject alkaline earth divalent cations. PAGE separation of product bands from in-line probing assays wherein 5′ ^32^P-labelled 66 *kefB* RNA was incubated with various alkaline earth divalent cations. Annotations are as described for Fig. 2b. Note that only the Na^+^ control assay (includes 10 mM NaCl and 2 mM MgCl_2_) yields the banding pattern changes indicative of ligand binding. The other assays depicted were conducted with 2 mM MgCl_2_ supplemented with 10 mM of the divalent metal ions indicated, and these do not yield the same banding pattern changes exclusively observed upon the addition of Na^+^. Also note that some metal ions such as Mg^2+^ enhance the overall rate of spontaneous RNA cleavage as expected due to their ability to favor deprotonation of the RNA 2′ hydroxyl group.

**Extended Data Fig. 4:**
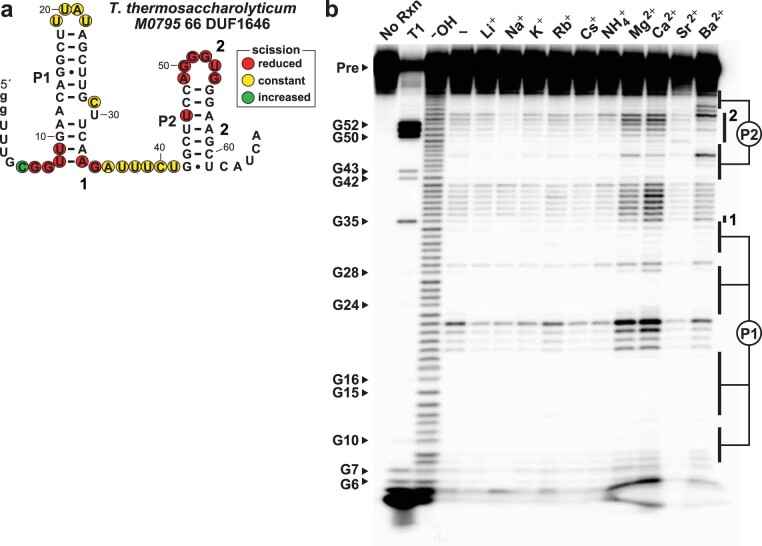
Ligand-binding specificity of a DUF1646 motif representative from *Thermoanaerobacterium thermosaccharolyticum* M0795. **a**, Sequence and secondary structure model for a DUF1646 representative from *T. thermosaccharolyticum* M0795 carrying 66 nucleotides and including two nonnative G nucleotides (gg) on the 5′ terminus to facilitate efficient production by in vitro transcription. **b**, Autoradiogram of PAGE-separated products resulting from in-line probing assays using the RNA construct depicted in a. In-line probing reactions were modified from the standard reactions conditions to exclude KCl and to contain low (2 mM) MgCl_2_. Monovalent ions as annotated for each lane were added to the reaction mixture at either 500 mM (Li^+^, Na^+^, Cs^+^, NH_4_^+^), 300 mM (K^+^), or 50 mM (Rb^+^). Divalent ions were tested at 10 mM. Notes: (*i*) Some divalent ions such as Mg^2+^ accelerate RNA strand scission in a concentration-dependent manner, and therefore these ions were not tested at higher concentrations. (*ii*) The lane with Sr^2+^ appears to be loaded with less RNA, which results in a broad reduction of intensities of all bands. Regardless, the lack of selective band modulation suggests that Sr^2+^ is not specifically bound by the RNA.

Furthermore, all alkaline earth divalent cations tested (Fig. 1c) fail to trigger banding pattern changes such as those observed when Na^+^ is added to in-line probing reactions with the 66 *kefB* RNA (Extended Data Fig. 3) or with a DUF1646 construct derived from another bacterial species (Extended Data Fig. 4). Thus, the distinctive RNA shape changes observed for DUF1646 motif RNAs are selectively triggered by Na^+^ binding, rather than occurring as an outcome of simple ionic strength increases.

Also consistent with Na^+^ aptamer function is the fact that mutations to highly conserved nucleotides of the wild-type (WT) 66 *kefB* RNA (Fig. 2a) disrupt the structural modulation induced by Na^+^ addition. Constructs carrying mutations at strictly conserved nucleotide positions at the base of P1 (construct M1, A32U and A33U) or in the loop of P2 (construct M2, G50U and G51U), cause a complete loss of the distinctive Na^+^-dependent banding pattern changes observed from in-line probing assays (Fig. 2e). Thus, the conserved nucleotides that define DUF1646 motif RNAs are required for members of this class to selectively bind Na^+^.

### A Na^+^ riboswitch regulates transcription termination

**Fig. 3:**
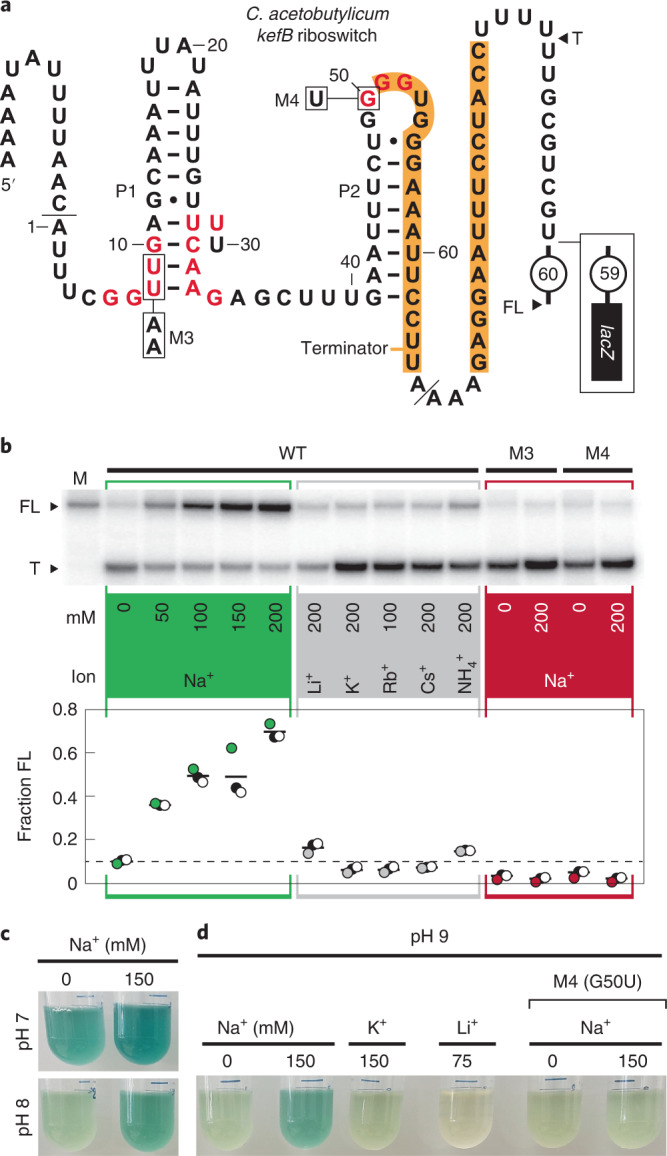
Na^+^ selectively triggers riboswitch-mediated transcription read-through and increased gene expression. **a**, WT Na^+^ riboswitch model based on the DUF1646 motif representative from the *C. acetobutylicum kefB* gene. Approximate 3′ termini for the terminated (T) and FL RNA transcripts are indicated. FL carries an additional 60 nucleotides (encircled number) not shown. The genetic reporter construct carries different 3′ nucleotides, including the *lacZ* gene. Additional annotations are as describe for Figs. 1 and 2. **b**, Top, representative PAGE analysis of in vitro transcription termination assays. Bands corresponding to the T and FL RNA products as defined in **a** are indicated. Lane M (marker) was loaded with a transcript corresponding to FL RNA. Bottom, a plot of the fraction of FL transcripts versus ion supplementation for various constructs. Data points (*n* = 3) corresponding to the PAGE autoradiogram depicted are coloured the same as the annotation panels. Open and black-filled data points were derived from two additional replicate assays (autoradiograms not shown). Solid lines represent average values for each condition, and the dashed line represents the average value for the WT construct with no Na^+^ supplementation. All transcription reactions include at least 4 mM Na^+^ due to its presence in the standard transcription reaction buffer, and this is not reflected in the ion identities and concentrations presented in the graphic. **c**, Representative reporter assays using surrogate *B. subtilis* cells carrying the riboswitch–reporter construct depicted in **a**. Cells were cultured in low-sodium LB media at the indicated pH value and containing roughly 15 mM Na^+^ and X-gal, which was used either without alteration (–) or was supplemented with 150 mM NaCl (Extended Data Fig. 5 and Supplementary Table 1). See Methods for additional details. **d**, Representative reporter assays conducted as described in **c** using WT or M4 riboswitch reporters cultured in low-sodium LB medium at pH 9, used without alteration (–) or supplementation with the monovalent ions indicated (Extended Data Fig. 5 and Supplementary Table 1). Lower Li^+^ concentration was used to avoid strong growth inhibition. Source data

The 66 *kefB* RNA sequence naturally resides a short distance upstream of six contiguous U nucleotides, which is characteristic of a transcriptional pause sequence for an intrinsic transcription terminator stem^31^. The RNA can potentially form a strong stem (Fig. 3a, orange shading) immediately upstream of the U nucleotides, although this putative terminator stem can form only at the expense of the P2 stem of the Na^+^ aptamer. This structural versatility suggests that the riboswitch uses a terminator stem as part of its gene-regulating ‘expression platform’^1^. The mutually exclusive formation of aptamer and terminator stem structures is a common mechanism by which riboswitches convert a ligand-binding event into a change in gene expression^3^. If this model is correct, Na^+^ binding by the aptamer reinforces P2 formation and precludes the formation of the terminator stem. This architecture, which is also observed with other DUF1646 motif representatives^20^, is consistent with our hypothesis that the RNA functions as a genetic ON switch wherein Na^+^ promotes transcription of the full-length (FL) mRNA.

In vitro transcription assays were used to determine whether the riboswitch regulates transcription elongation in a Na^+^-dependent manner. A double-stranded DNA construct carrying a constitutive promoter was used as a template for the transcription of an extended version of the 66 *kefB* RNA construct (Fig. 3a) using *E. coli* RNA polymerase holoenzyme. The resulting RNA carries 13 additional nucleotides relative to 66 *kefB* on the 5′ end to facilitate in vitro, single-round transcription. The FL RNA also includes 91 additional nucleotides on the 3′ terminus, which encompasses the natural expression platform for the putative riboswitch.

Two possible RNA transcripts, called terminated or FL, are expected when the corresponding DNA construct is subjected to transcription. The terminated product of roughly 103 nucleotides is formed if the intrinsic terminator halts transcription within the run of U nucleotides (Fig. 3a, terminated). However, if the terminator stem is blocked from forming, as predicted to occur when Na^+^ is bound by the aptamer, then the RNA transcript is expected to be roughly 170 nucleotides (Fig. 3a, FL). Indeed, the addition of progressively higher concentrations of Na^+^ to transcription reactions causes the WT construct to yield greater amounts of the FL product (Fig. 3b). In contrast, various alkali cations, or ammonium, fail to induce transcription past the terminator stem, which is consistent with the failure of these ions to trigger RNA structure changes as determined by in-line probing. DNA templates carrying mutations to highly conserved nucleotides of the Na^+^ aptamer (Fig. 3a, M3 and M4) cause a complete loss of Na^+^-dependent transcriptional read-through (Fig. 3b). These results demonstrate a mechanism by which DUF1646 RNAs can sense Na^+^ and regulate genes relevant to sodium biology.

### Na^+^ riboswitch regulation of gene expression in bacteria

**Extended Data Fig. 5:**
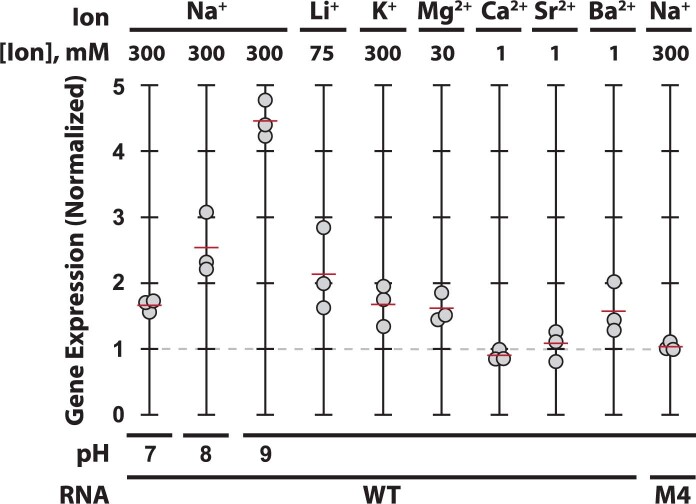
Quantitation of riboswitch-reporter fusion assays. *B. subtilis* cells carrying the WT or M4 riboswitch-reporter constructs depicted in Fig. 3a were grown overnight in low-sodium (~15 mM Na^+^) LB (yeast extract and tryptone). 20 µL of the overnight culture was subcultured in low-sodium LB buffered at pH 7.0 (100 mM PIPES), pH 8.0 (100 mM TAPS), or pH 9.0 (100 mM AMPSO), all containing chloramphenicol (5 µg/mL). As indicated for each assay, ions were added to supplement the media, the mixtures were incubated overnight, and ONPG (ortho-nitrophenyl-ß-galactoside) was added to samples to measure β-galactosidase activity (units presented in Supplementary Table 1) by adapting the method described previously [Miller, J.H. Procedures for working with lac. In: A Short Course in Bacterial Genetics. (Cold Spring Harbor, NY: Cold Spring Harbor Laboratory Press) pp 72 (1992)]. Three independent replicates of bacterial cultures and gene expression assays were performed for each condition. Depicted are plots of gene expression normalized by setting the low Na^+^ β-galactosidase activity units to 1 (dashed line). Red lines represent the mean for the three independent experiments. See Materials and Methods for additional details. Notes: Li^+^ and Mg^2+^ concentrations were chosen to avoid strong growth inhibition. Due to the poor solubility of Ca^2+^, Sr^2+^, and Ba^2+^ at pH 9.0, 1 mM concentrations were used because 10 mM concentrations in LB were observed to precipitate.

A genetic reporter-fusion construct was created by fusing the *kefB* riboswitch region to a *lacZ* (β-galactosidase) reporter gene (Fig. 3a, box), and the resulting construct was integrated into the *amyE* locus of the *Bacillus subtilis* chromosome for analysis. The WT riboswitch–reporter fusion construct yields the highest gene expression when cells are grown at pH 7 in rich medium (Luria-Bertani (LB)) supplemented with 150 mM NaCl (total [Na^+^] is around 165 mM) (Fig. 3c, Extended Data Fig. 5 and Supplementary Table 1). Cells grown in LB media lacking supplementation with Na^+^ (around 15 mM [Na^+^]), exhibit slightly lower gene expression.

The effect of Na^+^ on reporter gene expression is more pronounced when cells are grown at higher pH (Fig. 3c,d). *B. subtilis* is known to expel Na^+^ to transport H^+^ into cells, thereby maintaining internal pH within physiological limits^32,33^. Thus, in alkaline media the cellular concentration of Na^+^ is expected to be substantially reduced. Without Na^+^, the riboswitch adopts the competing terminator stem structure and *lacZ* mRNA transcription is halted. Supplementation with an additional 150 mM Na^+^ activates reporter gene expression, indicating that abundant Na^+^ in the medium enters the cells to offset the loss from their effort to maintain internal pH. However, reporter expression is not observed on addition of either K^+^ or Li^+^, or when the riboswitch carrying the M4 disabling mutation is used (Fig. 3d, Extended Data Fig. 5 and Supplementary Table 1). Again, these results are consistent with our hypothesis that DUF1646 motif RNAs function as aptamer components of Na^+^ riboswitches that activate gene expression when bound to their target metal ion.

### A natural Boolean logic gate for Na^+^ and c-di-AMP

In rare instances (Supplementary Table 2), a riboswitch for c-di-AMP^34^ is found immediately downstream of a Na^+^ riboswitch^20^. c-di-AMP regulates processes related to osmotic stress responses^34–36^, including those that are important for alkali metal homeostasis^37^. Riboswitches for this bacterial second messenger are known to selectively bind two c-di-AMP molecules^38^ and regulate the expression of genes commonly involved in osmotic shock mitigation and cell wall remodeling processes^34–36^. Therefore, we speculated that the tandem architecture of Na^+^ and c-di-AMP riboswitches might function as a two-input Boolean logic gate as is observed for certain other riboswitch classes^39,40^.

**Fig. 4:**
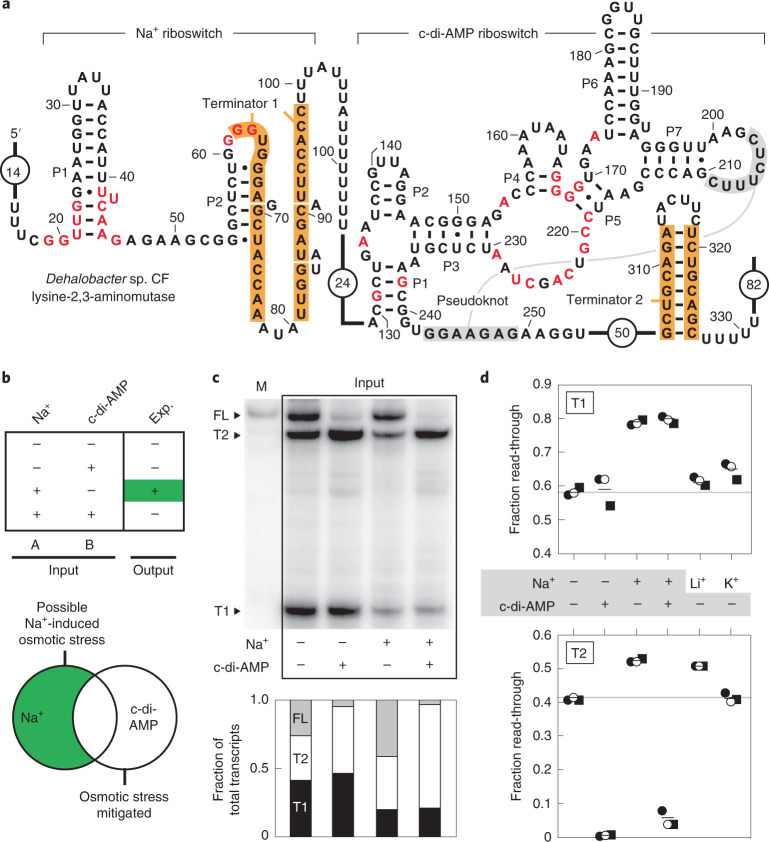
A natural tandem arrangement between riboswitches for Na^+^ and c-di-AMP operates as a two-input Boolean logic gate. **a**, Sequence and secondary structure model for the tandem riboswitch system associated with the lysine-2,3-aminomutase gene of *Dehalobacter* sp. CF. **b**, Top, a truth table for an A and Not B (material nonimplication) Boolean logic gate. The expected (exp.) output represents the gene expression outcome for an individual RNA where the riboswitches operate perfectly. Green represents active gene expression. Bottom, the expected gene expression trends as influenced by biological conditions. **c**, Top, in vitro transcription assays demonstrating independent operation of each riboswitch. T1, T2 and FL identify bands corresponding to RNAs ending at terminator 1, terminator 2, or reaching full length, respectively. Other annotations are as described for Fig. 3b. Bottom, a plot of the fraction of total transcripts terminating at sites T1, T2 and FL as depicted in the gel lanes above. Values were established by measuring band intensities and adjusting for the number of radiolabelled U nucleotides (Methods). **d**, Plots of the total fraction of in vitro transcription products proceeding past T1 (top) or T2 (bottom). Values from replicate experiments (*n* = 3) are represented. Solid lines represent the average values for each condition and gray lines represent the average value in the absence of Na^+^ and c-di-AMP inputs for comparison. Source data

Based on the tandem architecture (Fig. 4a), we predicted that the system functions as a genetic ‘A and Not B’ (material nonimplication or abjunction) logic gate (Fig. 4b)^41^. Each aptamer is immediately followed by a terminator stem, suggesting that the two aptamers are unlikely to interact with each other, but will function independently. Elevated Na^+^ should promote transcription beyond the first intrinsic terminator stem (T1), which favors the expression of a gene whose protein product generates the precursor for the osmolyte *N*^ε^-acetyl-β-lysine^42,43^. In contrast, elevated c-di-AMP concentrations signal to the cell that it is not in osmotic distress^36^, perhaps because the ionic imbalance has already been overcome by using a different osmolyte. In this latter situation, the c-di-AMP riboswitch is predicted to trigger transcription termination at the second terminator stem (T2), thereby preventing the unnecessary expression of the osmotic stress response gene even when Na^+^ concentrations are abnormally high.

**Extended Data Fig. 6:**
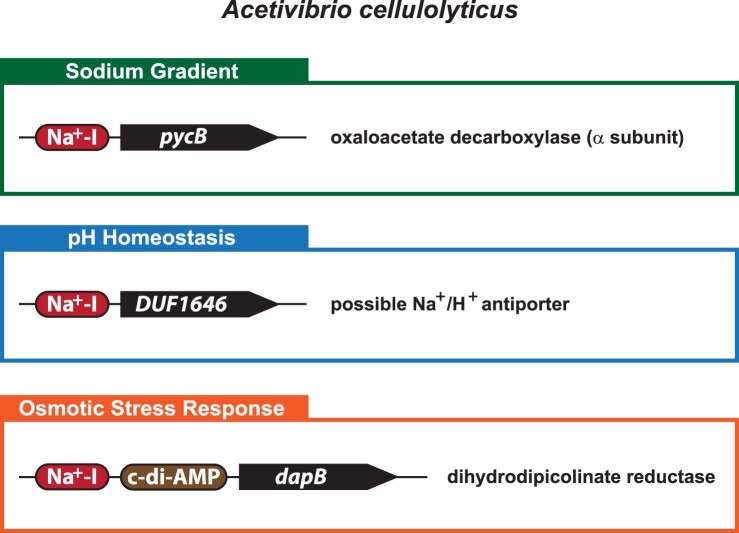
Three Na^+^ riboswitches in *Acetivibrio cellulolyticus* regulate three major processes related to sodium biology. The bacterium *A. cellulolyticus* carries three Na^+^-I riboswitch representatives (red) that regulate genes associated with three major aspects of sodium biology. *Sodium gradient:* The *pycB* gene codes for oxaloacetate decarboxylase, whose activity drives Na^+^ export to yield a Na^+^ gradient [Dimroth, P., Jockel, P. & Schmid, M. Coupling mechanism of the oxaloacetate decarboxylase Na^+^ pump. *Biochim. Biophys. Acta Bioenerg*. **1505**, 1-14 (2001)]. The gradient can then be exploited to promote solute uptake by Na^+^-dependent import proteins [Pos, K. M. & Dimroth, P. Functional properties of the purified Na^+^-dependent citrate carrier of *Klebsiella pneumoniae*: evidence for asymmetric orientation of the carrier protein in proteoliposomes. *Biochemistry* **35**, 1018-1026 (1996)]. *pH homeostasis:* DUF1646 genes code for proteins of unknown function but some have been predicted to function as a Na^+^/H^+^ antiporters [CDD Conserved Protein Domain Family: DUF1646 (nih.gov); https://www.genome.jp/dbget-bin/www_bget?pop:7465038], which presumably can be used to increase the pH of cells growing under alkaline conditions. Osmotic stress response: Tandem Na^+^-I and c-di-AMP (brown) riboswitches are associated with *dapB*, which codes for dihydrodipicolinate reductase. This enzyme produces 2,3,4,5-tetrahydrodiaminopimelate, which is a precursor for both lysine and peptidoglycan biosynthesis [https://www.genome.jp/pathway/lin00300+dapB]. Peptidoglycan biosynthesis is critical for cell resistance to osmotic stress in high salt environments [Auer, G. K. & Weibel, D. B. Bacterial cell mechanics. *Biochemistry* **56**, 3710-3724 (2017); Metris, A., George, S. M., Mulholland, F., Carter, A. T. & Baranyi, J. Metabolic shift of *Escherichia coli* under salt stress in the presence of glycine betaine. *Appl. Environ. Microb*. **80**, 4745-4756 (2014)]. *E. coli* cells grown in 4.5% NaCl exhibit increased expression of *dapB*, and our findings indicate that some species directly regulate this gene using a Na^+^-responsive tandem riboswitch.

To evaluate this hypothesis, we examined a tandem Na^+^ and c-di-AMP riboswitch system from *Dehalobacter* sp. CF using in vitro transcription assays. As predicted, each riboswitch responds to its cognate ligand independently (Fig. 4c,d) to favor the proposed genetic output (Fig. 4b). Specifically, Na^+^ promotes read-through of T1 to roughly 80%, thereby yielding the greatest amount of FL RNA transcripts. In contrast, c-di-AMP permits less than 5% read-through to yield FL RNA. Although the populations of RNA transcripts do not reflect perfect logic gate outputs, the transcription termination trends observed in our assays are consistent with the hypothesis that the riboswitch logic gate system evaluates the concentrations of two ligands as an A and Not B logic gate and adjusts the expression of a gene related to osmolyte production (Extended Data Fig. 6). Unless osmotic stress conditions in high Na^+^ concentrations activate c-di-AMP phosphodiesterases that degrade the second messenger^36^, the Na^+^ riboswitch decision to produce more FL mRNA is strongly overridden by using the c-di-AMP riboswitch to turn off gene expression. The extent to which cells approach perfect logic gate function presumably is affected by the concentrations of the riboswitch ligands and possibly other cellular conditions or evolutionary adaptations not represented in our in vitro experiments.

## Discussion

Given the importance of Na^+^ to living systems, it seems likely that most species will carry sensors for this alkali metal ion that evaluate its concentration and regulate the expression of genes relevant to Na^+^ homeostasis or exploitation. The discovery and experimental validation of a riboswitch class that selectively responds to Na^+^ helps address the puzzling observation that only one experimentally validated protein factor, NhaR, has been proved to function as a Na^+^-responsive gene control factor^18^.

Our bioinformatic searches revealed a total of 308 distinct representatives of the DUF1646 motif, which makes this riboswitch relatively rare compared to many other riboswitch classes^1,2^. Therefore, we expect that many additional Na^+^-sensing gene control factors exist in living systems that await discovery. Indeed, we have determined that rare variants of a similar orphan riboswitch candidate class originally called the *nhaA*-I motif^20^ also sense Na^+^ and regulate gene expression^44^. Thus, we propose naming DUF1646 motif RNAs as Na^+^-I riboswitches and the variant *nhaA*-I motif RNAs as Na^+^-II riboswitches.

Most riboswitch aptamers must be selective for their target ligands to avoid accidental regulation by close chemical analogs that might be present in cells. However, the genes associated with DUF1646 motif RNAs are sometimes annotated as encoding protein products that are relevant to other cations (Fig. 1b). This introduced some initial uncertainty regarding our hypothesis that these RNAs function as components of Na^+^-sensing riboswitches. Although all biochemical and genetic analyses conducted in the current study are consistent with Na^+^ as the riboswitch ligand, we cannot rule out the possibility that some representatives of the DUF1646 motif might have acquired mutations that alter their ligand specificity.

It remains unclear how Na^+^ riboswitches form selective binding pockets that exclude other alkali and alkaline earth cations. One factor that could be exploited is ionic radius, which differs widely among these cations (Fig. 1c). However, the ionic radius of Na^+^ and Ca^2+^ are nearly identical, and therefore binding pocket size cannot fully explain ligand selectivity. Perhaps the binding pocket in Na^+^ riboswitches cannot stably accommodate the two positive charges of Ca^2+^. Furthermore, in protein structures, Na^+^ prefers to interact with oxygen nuclei, exhibits distinct bonding distances and forms coordination geometries that can be different from those of other cations^45^. Future structural analysis studies should reveal how Na^+^ RNA aptamers can form selective binding pockets for this cation.

Some genes associated with Na^+^ riboswitches (Fig. 1b) express proteins that are annotated with functions that are not obviously related to sodium biology, such as those coding for transporters of K^+^ (KefB) and Mg^2+^ (MgtA), which could be relevant to a cell’s response to sodium-triggered osmotic stress. However, many gene products are annotated based on sequence similarity to proteins with experimentally validated functions, but these automated predictions are not always correct. Thus, the riboswitch-associated ion transporters noted above could be selective for Na^+^ rather than the ions indicated by the gene annotations.

The names of some other Na^+^-riboswitch-associated genes are both compelling and intriguing. In addition to genes related to Na^+^ homeostasis and osmotic stress responses, some genes code for ATP synthase components. This provides links between Na^+^ sensing and the exploitation of Na^+^ gradients for energy generation. All three of these processes related to sodium biology are present in the bacterium *Acetivibrio cellulolyticus*, which regulates the relevant genes by using three different Na^+^ riboswitch representatives (Extended Data Fig. 6).

The tandem arrangement of Na^+^ and c-di-AMP riboswitches observed in *Dehalobacter* sp. CF (Fig. 4a) provides the host cell with the ability to integrate two fundamental signals to appropriately adjust gene expression for the current cellular environment. The presence of an intrinsic terminator stem associated with each aptamer indicates that the two riboswitches function independently. The Na^+^ aptamer overlaps with the right shoulder of the predicted terminator stem, and thus was predicted to operate as a genetic ON switch, wherein cation binding precludes the formation of the terminator to allow transcription to proceed beyond T1. In contrast, the c-di-AMP aptamer resides apart from the intrinsic terminator stem and thus was predicted to function as a genetic OFF switch, wherein binding of the second messenger causes transcription termination.

Consistent with this last prediction is the fact that nucleotides 238–243 of the c-di-AMP aptamer, including some that form the right shoulder of P1 and the aptamer pseudoknot, can form an alternative base-pairing interaction with nucleotides 304–309 that form the left shoulder of the terminator stem. Thus, nucleotides in the core of the aptamer are predicted to interfere with terminator stem formation only when c-di-AMP is absent.

Abnormally high Na^+^ concentration in cells may be indicative of a high salt environment that places the cells under osmotic stress. This would explain why Na^+^ riboswitches are occasionally associated with genes coding for proteins that increase the concentrations of osmoprotectants or their precursors, including *dapB* (lysine), *ablA* (β-lysine) and *eam* (β-glutamate). The gene associated with the tandem riboswitch from *Dehalobacter* sp. CF is lysine-2,3-aminomutase (*ablA*), which catalyses a reaction that converts lysine into β-lysine. This compound is a known osmoprotectant in archaea^42^ and bacteria^43^.

Many bacteria exploit a sodium ion cycle^12^ wherein they exploit a Na^+^ gradient to drive solute transport^15^, or to power other biological processes. For example, some bacterial species are known to exploit Na^+^ gradients to generate ATP^17,26,46^. Our findings reveal that DUF1646 motif RNAs (Na^+^-I riboswitches) are sometimes associated with genes coding for ATP synthase components, suggesting that species carrying these arrangements are using a riboswitch to evaluate cellular Na^+^ concentrations and regulate the exploitation of Na^+^ gradients to generate energy through the synthesis of ATP. It was previously known that a sodium-translocating V-type ATPase was regulated by Na^+^ concentrations^47^. We now observe that V-type ATPase genes from various species of enterococci and clostridia are associated with members of this Na^+^ riboswitch class, which provides a mechanism for selective regulation by Na^+^.

To complete the sodium ion cycle and reduce cellular Na^+^ concentration, or to reestablish a gradient across the membrane, some bacterial species eject Na^+^ by harnessing energy derived from the action of certain metabolic enzymes such as oxaloacetate decarboxylase. This enzyme, whose gene is occasionally associated with DUF1646 RNAs (Fig. 1b), is known to couple its decarboxylation activity with Na^+^ transport^25^. Additionally, many species exploit Na^+^/H^+^ antiporters that adjust cellular sodium concentrations. For example, the reporter gene assays conducted in this study (Fig. 3c,d) appear to be influenced by a known mechanism wherein Na^+^ is expelled in the process of maintaining the internal pH of cells residing in an alkaline environment^32,33^. Only at high pH do cells eject sufficient Na^+^ to turn off reporter gene expression regulated by the riboswitch examined in our study.

It has been speculated that the exploitation of Na^+^ gradients to form high-energy compounds is an ancient process that predated the exploitation of proton gradients^48^. We^1–3^ have described evidence that many riboswitch classes present in modern cells likely emerged during the RNA World, a time before the evolutionary dominance of protein enzymes and receptors^4^. The discovery of Na^+^-selective riboswitches reveals that RNA molecules indeed have the structural sophistication necessary to form selective binding pockets for Na^+^. Such molecules would have been necessary for ancient RNA World organisms to monitor and exploit sodium gradients, which would have been important if energy derivation involved the creation and use of these gradients.

Assuming that Na^+^ riboswitches will primarily associate with genes related to sodium biology, it is now possible to identify the larger collection of genes whose expression is regulated by cellular concentrations of cationic sodium. The identification of such ‘superregulons’ for other riboswitch classes, such as those for c-di-AMP^34^, has been helpful in identifying proteins involved in new signaling or metabolic pathways. Thus, we anticipate that further efforts to identify genes associated with Na^+^-I riboswitches will help researchers expand the known collection of genes associated with Na^+^ homeostasis and exploitation.

Our results also highlight a common problem encountered when seeking to validate the functions of riboswitches. As noted above, DUF1646 RNAs are frequently associated with genes whose protein products are directly related to Na^+^ biology. However, some associated genes are annotated as transporters of potassium or magnesium (Fig. 1b). For example, one of the most common genes associated with DUF1646 RNAs is annotated as *kefB*^20^, including the gene associated with the *C. acetobutylicum* riboswitch RNA we examined in the current study (Figs. 2 and 3). Some KefB proteins have indeed been proved to promote K^+^ transport^49^. However, it seems most likely that the proteins with genes associated with Na^+^ riboswitches have adapted to function as Na^+^ transporters. This speculation is supported by the fact that the *kefB*-derived riboswitch examined herein only responds to Na^+^, and strongly rejects K^+^ in in-line probing assays (Fig. 2d), transcription termination assays (Fig. 3b) and in riboswitch–reporter fusion assays (Fig. 3c,d). Efforts to examine gene associations therefore might help assign correct biochemical functions to the proteins encoded by genes associated with the riboswitch. For example, it seems possible that genes annotated as K^+^ or Mg^2+^ transporters might actually transport Na^+^.

## Methods

### Chemicals and biochemicals

Lithium chloride was purchased from Acros Organics. Ammonium chloride was purchased from Macron Fine Chemicals. Calcium chloride dihydrate and magnesium chloride hexahydrate were purchased from J.T. Baker. All salts were 99% pure or greater, except calcium chloride dihydrate was 97% or greater. [γ-^32^P]ATP and [α-^32^P]UTP were purchased from PerkinElmer. All other chemicals and synthetic DNA oligonucleotides were purchased from Sigma-Aldrich. RNase T1 was purchased from Roche. All other enzymes were purchased from New England Biolabs unless otherwise indicated.

### Bioinformatics analyses

The initial consensus sequence and secondary structure model for DUF1646 motif RNAs^24^ was updated by searching for additional representatives using CMfinder^51^ and Infernal 1.1 (ref. ^52^) as previously described^24^. The databases examined included genomic DNA sequences from the National Center for Biotechnology Information Reference Sequence Database v.80 as well as microbial environmental sequences collections (env12). A total of 308 nonredundant DUF1646 motif RNA representatives were used to produce an updated consensus sequence and secondary structure model (Fig. 1a) that was generated using the computer program R2R (ref. ^53^). Covariation annotations were further assessed using R-scape^54^.

Gene association data (first gene located immediately downstream of each representative) were established by manual analysis and by using protein sequence homology as established via the National Center for Biotechnology Information Basic Local Alignment Search Tool (ref. ^55^). The resulting list of gene annotations was organized to generate a pie chart (Fig. 1b).

### RNA construct preparation

DNA templates for RNA transcription were generated through extension of overlapping synthetic DNA oligonucleotides using SuperScript II reverse transcriptase following the manufacturer’s directions (Thermo Fisher Scientific). Each DNA template was transcribed with T7 RNA polymerase in 50 μl reactions (80 mM HEPES (pH 7.5 at roughly 20 °C), 24 mM MgCl_2_, 2 mM spermidine, 40 mM dithiothreitol (DTT)) incubated overnight at 37 °C. Resulting RNA products were separated via denaturing (8 M urea) PAGE. Bands containing the desired FL RNAs were excised, crushed and incubated in 350 μl of crush-soak solution (200 mM NH_4_Cl, 10 mM Tris-HCl (pH 7.5 at around 20 °C), 1 mM EDTA) for 30 min at around 20 °C. Purified RNAs were precipitated by the addition of 850 μl of cold ethanol, centrifuged to pellet, dried under vacuum and resuspended in 25 μl of deionized water (dH_2_O). The concentration of each RNA solution was estimated by measuring the absorbance at 260 nm and calculating its molarity using the estimated extinction coefficient of the construct.

Next, 75 pmol of each RNA was dephosphorylated using rAPid Alkaline Phosphatase (Roche) following the manufacturer’s protocol. Then 10 pmol of RNA was 5′ ^32^P-labelled using T4 polynucleotide kinase in a 20 μl reaction containing 5 mM MgCl_2_, 25 mM CHES (N-Cyclohexyl-2-aminoethanesulfonic acid) (pH 9.0), 3 mM DTT and 20 μCi [γ-^32^P]-ATP. Radiolabelled RNA was purified as described above, and salts were removed by performing three dH_2_O washes through an Amicon Ultra-0.5 centrifugal filter unit (3 kDa molecular weight cutoff).

### In-line probing assays

In-line probing^32,33^ is a method typically used for establishing the ligand-binding characteristics of riboswitch aptamers. This assay takes advantage of the fact that most aptamers undergo a shape change in response to ligand binding, which can be evaluated based on changes in the speed at which RNA internucleotide linkages undergo spontaneous breakdown^56^. Specifically, linkages within unstructured regions of an RNA molecule can experience nucleophilic attack by a 2′ oxygen atom on its adjacent phosphorus center to cause internal phosphoester transfer and RNA chain cleavage. The addition of the target ligand causes the RNA aptamer to undergo structural changes that alter the pattern of spontaneous RNA strand scission, and these sites can be mapped to establish various structural and functional characteristics of a riboswitch.

In-line probing assays were conducted generally as described previously^32,33^, except that the concentration of MgCl_2_ was reduced to 2 mM and the concentrations of monovalent ions were specifically adjusted. Desalted, radiolabelled RNAs were incubated with candidate ligands for 1 min at 75 °C before the addition of low-salt in-line probing buffer (50 mM Tris-HCl (pH 8.3 at approximately 20 °C), 2 mM MgCl_2_, plus or minus monovalent ions as indicated for each experiment) and incubation at room temperature for between 40 and 70 h. RNA degradation products were separated by denaturing 10% PAGE and visualized using a Typhoon FLA 9500 Molecular Scanner (GE Healthcare). Dissociation constants for RNA–ligand interactions were estimated by quantifying changes in the band intensities corresponding to specific sites in the RNA structure that are modulated on varying the ligand concentration in individual reactions. Band intensities were quantified using ImageQuant software and normalized relative to a constant-intensity band. The resulting values were scaled to a fraction between 0 and 1 (greatest change), then plotted against the logarithm of the ligand concentration. Apparent *K*_D_ values were calculated using a sigmoidal-dose response equation in GraphPad Prism 8.

### In vitro transcription termination assays

For single Na^+^ riboswitch constructs, transcription termination assays were conducted by adapting a previously established method for single-round transcription assays^57^. DNA templates (Supplementary Table 3) for the *C. acetobytylicum* WT and M4 constructs (Fig. 3a) include the native promoter. Transcription reactions were performed with 100 nM DNA template in 40 mM Tris-HCl (pH 7.5 at 20 °C), 200 mM KCl, 2 mM MgCl_2_, 0.01 mg ml^−1^ BSA, 1% v/v glycerol and 0.04 U µl^−1^ *E. coli* RNA polymerase (New England Biolabs). RNA polymerase is supplied in a buffer that adds roughly 4 mM NaCl to the final reaction. The dinucleotide, ApA (0.135 mM), was added to the reaction for initiation by RNA polymerase. The nucleotides guanosine triphosphate (GTP) and ATP (2.5 µM each), uridine triphosphate (UTP) (1.0 µM) and [α-^32^P]-UTP (2 µCi) were also added. The resulting reaction mixture was initially incubated for 10 min at 37 °C, during which RNA polymerase is expected to stall at the first C residue, which occurs 13 nucleotides from the transcription start site. The halted complexes resumed transcription with the addition of 150 µM each of GTP, ATP and cytidine triphosphate and 30 µM UTP. Also, 0.1 mg ml^−1^ heparin was added at that time to prevent further transcription initiation and the mixture was incubated for an additional 30 min at 37 °C.

Monovalent ions (potential riboswitch ligands) were supplemented in the elongation mix after the stable initiation complex was formed to reduce effects on transcription yield. Additional monovalent ions were included in the transcription reactions as annotated for each experiment. However, all reactions also include and additional roughly 4 mM Na^+^ as noted above plus 200 mM KCl that are not reflected by the annotations. For example, the reaction annotated as supplemented with 200 mM K^+^ (Fig. 3b) actually has 400 mM K^+^ and roughly 4 mM Na^+^. Products were separated by denaturing 10% PAGE, imaged using a PhosphorImager and quantified using ImageQuant software.

Three replicates were performed on three different days, and a representative autoradiogram from PAGE gel separation of the products is depicted (Fig. 3b, top). The percentage of [α-^32^P]-UTP compared to total UTP concentration in the initiation and elongation reactions (7 and 0.2%, respectively) was used to convert measured radioactivity values to transcript values. Specifically, relative amount of radioactivity per terminated (*R*_T_) and FL (*R*_FL_) transcripts was calculated for each transcript size using the following equation: ((number of U residues in initiation region)(7%)) + ((number of U residues in elongation region)(0.2%)) = *R*. *R*_T_/*R*_FL_ is equal to the correction factor (X%) that accounts for the increased number of radiolabelled U residues in the FL transcript. The equation used to establish the percentage of transcription termination was: 100(*T*/(*T* + (FL)(X%))) = percentage termination. These values were divided by 100 to yield values for the fraction of FL RNAs.

For tandem Na^+^ riboswitch and c-di-AMP riboswitch constructs, transcription termination assays were conducted generally as described above. The DNA template (Supplementary Table 3) carried the 5ʹ UTR from *Dehalobacter* sp. CF, which contains 18 nucleotides upstream of the first highly conserved (≥97%) residue plus the entire Na^+^ and c-di-AMP riboswitches. The native G at the 14th position (in the nonconserved region) was converted to a C to create a transcriptional halt site. The promoter from the *B. subtilis lysC* gene was used to drive transcription by *E. coli* RNA polymerase holoenzyme as described previously^58^.

Tandem transcription reactions were performed with 100 nM DNA template in 40 mM Tris-HCl (pH 7.5 at 20 °C), 100 mM KCl, 4 mM MgCl_2_, 0.01 mg ml^−1^ BSA, 1% v/v glycerol and 0.04 U µl^−1^ *E. coli* RNA polymerase (New England Biolabs). Transcription reactions did not include the dinucleotide ApA because c-di-AMP riboswitches are known to recognize ApA^38^. GTP and ATP (5 µM each), UTP (2.0 µM) and [α-^32^P]-UTP (2 µCi) were included, and the reaction mixture was initially incubated for 10 min at 37 °C. The halted complexes were triggered to resume transcription on addition of 150 µM each of GTP, ATP and cytidine triphosphate and 15 µM UTP. Again, 0.1 mg ml^−1^ heparin was added, and the mixture was incubated for an additional 30 min at 37 °C. Products were separated and analysed as described above.

Three replicate assays were performed on different days for each experiment. A representative autoradiogram is shown (Fig. 4c) to illustrate the effects of Na^+^ and c-di-AMP on the tandem riboswitch system. The percentage of [α-^32^P]-UTP compared to total UTP concentration in the initiation and elongation reactions (3 and 0.4%, respectively) was used to compute the amounts of RNA transcripts for each product. Specifically, the amounts of RNA terminated at the Na^+^ riboswitch (*R*_T1_), terminated at the c-di-AMP riboswitch (*R*_T2_) and FL (*R*_FL_) were determined and used to generate plots (Fig. 4c, bottom and Fig. 4d). This was achieved using the following equation: ((number of U residues in initiation region)(3%)) + ((number of U residues in elongation region)(0.3%)) = *R*. *R*_T1_/((*R*_T2_)(*R*_FL_)) is equal to the correction factor (*X*_T2_%) that accounts for the increased number of radiolabelled U residues when termination occurs with the c-di-AMP riboswitch and (*X*_FL_%) that accounts for the increased number of radiolabelled U residues when the FL transcript is produced. The equation used to establish the percentage of transcription termination was: 100(T1/(T1 + (T2)(*X*_T2_%) + (FL)(*X*_FL_%))) = percentage termination. For determining the percentage terminated at the c-di-AMP riboswitch the following equation was used: percentage terminated = 100(T2/(T2 + (FL)(*X*_FL_%))). These values were divided by 100 to yield values for the fraction of FL RNAs. By using the fraction read-through at the DUF1646 terminator, that value was then subdivided using the fraction terminated at the c-di-AMP terminator to yield the fraction of each of the three possible transcripts.

### Reporter gene assays

The riboswitch–reporter fusion constructs were prepared as previously described^58,59^. Constructs were designed based on the *C. acetobutylicum* riboswitch representative used for the transcription termination assays (Fig. 3). Synthetic oligonucleotides were designed to carry the same native promoter and riboswitch sequences as those present in the transcription termination constructs. However, the synthetic DNAs for the riboswitch–reporter constructs encompass nucleotides ending 25 nucleotides upstream of the natural start codon. Synthetic oligonucleotides for the DUF1646 WT and M4 genetic constructs were amplified by PCR, prepared as an *EcoRI*-*BamHI* fragment, cloned into plasmid pDG1661 immediately upstream of the *lacZ* reporter gene, and confirmed by sequencing. The plasmid was then integrated into the *amyE* locus of *B. subtilis* strain 1A1 obtained from the *Bacillus* Genetic Stock Center (BGSC.org) and the desired genetic variants were selected by their resistance to chloramphenicol.

Genetic assays were conducted by first growing cells overnight in LB media. LB carries approximately 15 mM Na^+^, which is introduced from tryptone and yeast extract. Subsequently, 100 µl of overnight culture was transferred to 2 ml of fresh, LB media buffered at pH 7.0 (100 mM PIPES), pH 8.0 (100 mM TAPS) or pH 9.0 (100 mM AMPSO) (pH adjusted with KOH in all cases), containing X-gal (100 µg ml^−1^), chloramphenicol (5 µg ml) and supplemented with the cations indicated for each experiment. Buffers for media preparation were chosen based on established methods^60^. Monovalent ions were then added as indicated for each experiment. Cultures were incubated overnight and tubes were photographed to visualize β-galactosidase activity.

### Reporting summary

Further information on research design is available in the Nature Research Reporting Summary linked to this article.

## Online content

Any methods, additional references, Nature Research reporting summaries, source data, extended data, supplementary information, acknowledgements, peer review information; details of author contributions and competing interests; and statements of data and code availability are available at 10.1038/s41589-022-01086-4.

## Supplementary information

Supplementary Information Supplemental Tables 1–3.

Reporting Summary

## Data Availability

All data relevant to the conclusions are presented in the paper. Updated sequence alignments in sto format are available upon request. Source data are provided with this paper.

## Source data

Source Data Fig. 3 Uncropped gel from Fig. 3b.
Source Data Fig. 4 Uncropped gel from Fig. 4c.

## Extended data

are available for this paper at 10.1038/s41589-022-01086-4.

## Code availability

All computer codes are available as cited, and also will be sent upon request.

## References

[1] McCown PJ, Corbino KA, Stav S, Sherlock ME, Breaker RR (2017). Riboswitch diversity and distribution. RNA.

[2] Breaker, R. R. The biochemical landscape of riboswitch ligands. *Biochemistry* **61**, 137–149 (2022). 10.1021/acs.biochem.1c00765 PMC9971707 35068140

[3] Breaker RR (2012). Riboswitches and the RNA World. Cold Spring Harb. Perspect. Biol..

[4] Benner SA, Ellington AD, Tauer A (1989). Modern metabolism as a palimpsest of the RNA World. Proc. Natl Acad. Sci. USA.

[5] Cromie MJ, Shi Y, Latifi T, Groisman EA (2006). An RNA sensor for intracellular Mg^2+^. Cell.

[6] Dann CE (2007). Structure and mechanism of a metal-sensing regulatory RNA. Cell.

[7] Dambach, M. et al. The ubiquitous *yybP*-*ykoY* riboswitch is a manganese-responsive regulatory element. *Mol. Cell* **57**, 1099–1109 (2015). 10.1016/j.molcel.2015.01.035 PMC4376352 25794618

[8] Price IR, Gaballa A, Ding F, Helmann JD, Ke A (2015). Mn^2+^-sensing mechanisms of *yybP*-*ykoK* orphan riboswitches. Mol. Cell.

[9] Furukawa K (2015). Bacterial riboswitches cooperatively bind Ni^2+^ or Co^2+^ ions and control expression of heavy metal transporters. Mol. Cell.

[10] Xu J, Cotruvo JA (2020). The *czcD* (NiCo) riboswitch responds to iron(II). Biochemistry.

[11] Baker JL (2012). Widespread genetic switches and toxicity resistance proteins for fluoride. Science.

[12] Häse CC, Fedorova ND, Galperin MY (2001). Sodium ion cycle in bacterial pathogens: evidence from cross-genome comparisons. Microbiol. Mol. Biol. Rev..

[13] Padan E, Bibi E, Ito M, Krulwich TA (2005). Alkaline pH homeostasis in bacteria: new insights. BBA - Biomembranes.

[14] Wilson TH, Ding PZ (2001). Sodium-substrate cotransport in bacteria. BBA - Bioenerg..

[15] Castle AM, Macnab RM, Shulman RG (1986). Coupling between the sodium and proton gradients in respiring *Escherichia coli* cells measured by ^23^Na and ^31^P nuclear magnetic resonance. J. Biol. Chem..

[16] Padan E (2004). NhaA of *Escherichia coli*, as a model of a pH-regulated Na+/H+ antiporter. BBA - Bioenerg..

[17] Efiok BJS, Webster DA (1992). Sodium-coupled ATP synthesis in the bacterium. Vitreoscilla. Arch. Biochem. Biophys..

[18] Rahav-Manor O (1992). NhaR, a protein homologous to a family of bacterial regulatory proteins (LysR), regulates *nhaA*, the sodium proton antiporter gene in *Escherichia coli*. J. Biol. Chem..

[19] Richard H, Foster JW (2007). Sodium regulates *Escherichia coli* acid resistance, and influences GadX- and GadW-dependent activation of *gadE*. Microbiol..

[20] Weinberg Z (2017). Detection of 224 candidate structured RNAs by comparative analysis of specific subsets of intergenic regions. Nucleic Acids Res..

[21] Torabi S-F (2015). In vitro selection of a sodium-specific DNAzyme and its application in intracellular sensing. Proc. Natl Acad. Sci. USA.

[22] Zhou W, Ding J, Liu J (2016). A highly specific sodium aptamer probed by 2-aminopurine for robust Na^+^ sensing. Nucleic Acids Res..

[23] Serganov A, Huang L, Patel DJ (2008). Structural insights into amino acid binding and gene control by a lysine riboswitch. Nature.

[24] Lee C (2013). A two-domain elevator mechanism for sodium/proton antiport. Nature.

[25] Studer R (2007). Crystal structure of the carboxyltransferase domain of the oxaloacetate decarboxylase Na^+^ pump from *Vibrio cholerae*. J. Mol. Biol..

[26] Mulkidjanian AY, Dibrov P, Galperin MY (2008). The past and present of sodium energetics: may the sodium-motive force be with you. Biochim. Biophys. Acta, Bioenerg..

[27] Dar D (2016). Term-seq reveals abundant ribo-regulation of antibiotics resistance in bacteria. Science.

[28] Soukup GA, Breaker RR (1999). Relationship between internucleotide linkage geometry and the stability of RNA. RNA.

[29] Regulski EE, Breaker RR (2008). In-line probing analysis of riboswitches. Methods Mol. Biol..

[30] Stautz J (2021). Molecular mechanisms for bacterial potassium homeostasis. J. Mol. Biol..

[31] Yarnell WS, Roberts JW (1999). Mechanism of intrinsic transcription termination and antitermination. Science.

[32] Cheng JA, Guffanti A, Krulwich TA (1997). A two-gene ABC-type transport system that extrudes Na^+^ in *Bacillus subtilis* is induced by ethanol or protonophore. Mol. Microbiol..

[33] Ito M, Guffanti AA, Oudega B, Krulwich TA (1999). *mrp*, a multigene, multifunctional locus in *Bacillus subtilis* with roles in resistance to cholate and to Na^+^ and in pH homeostasis. J. Bacteriol..

[34] Nelson JW (2013). Riboswitches in eubacteria sense the second messenger c-di-AMP. Nat. Chem. Biol..

[35] Commichau FM, Gibhardt J, Halbedel S, Gundlach J, Stülke J (2018). A delicate connection: c-di-AMP affects cell integrity by controlling osmolyte transport. Trends Microbiol..

[36] Stülke J, Krüger L (2020). Cyclic-di-AMP signaling in bacteria. Annu. Rev. Microbiol..

[37] Gundlach J (2019). Sustained sensing in potassium homeostasis: cyclic di-AMP controls potassium uptake by KimA at the levels of expression and activity. J. Biol. Chem..

[38] Gao A, Serganov A (2014). Structural insights into recognition of c-di-AMP by the *ydaO* riboswitch. Nat. Chem. Biol..

[39] Sudarsan N (2006). Tandem riboswitch architectures exhibit complex gene control functions. Science.

[40] Sherlock ME, Sudarsan N, Stav S, Breaker RR (2018). Tandem riboswitches form a natural Boolean logic gate to control purine metabolism in bacteria. eLife.

[41] Lehman, E., Leighton, F. T. & Meyer, A. R. in *Mathematics for Computer Science* (12th Media Services, 2017).

[42] Pflüger K (2003). Lysine-2,3-aminomutase and β-lysine acetyltransferase genes of methanogenic archaea are salt induced and are essential for the biosynthesis of *N*^ε^-acetyl-β-lysine and growth at high salinity. Appl. Environ. Microbiol..

[43] Triadó-Margarit X, Vila X, Galinski EA (2011). Osmoadaptative accumulation of *N*^ε^-acetyl-β-lysine in green sulfur bacteria and *Bacillus cereus* CECT 148T. FEMS Microbiol. Lett..

[44] White, N., Sadeeshkumar, H., Sun. A., Sudarsan, N. & Breaker. R. R. Lithium-sensing riboswitch classes regulate expression of bacterial cation transporter genes (submitted). 10.1038/s41598-022-20695-6 PMC9646797 36352003

[45] Harding MM (2002). Metal-ligand geometry relevant to proteins and in proteins: sodium and potassium. Acta Crystallogr. D. Biol. Crystallogr..

[46] Schulz S (2013). A new type of Na^+^-driven ATP synthase membrane rotor with a two-carboxylate ion-coupling motif. PLoS Biol..

[47] Ikegami M, Kawano M, Takase K, Yamato I, Igarashi K (1999). *Enterococcus hirae* vacuolar ATPase is expressed in response to pH as well as sodium. FEBS Lett..

[48] Mulkidjanian AY, Galperin MY, Makarova KS, Wolf YI, Koonin EV (2008). Evolutionary primacy of sodium bioenergetics. Biol. Direct.

[49] Bakker EP, Booth IR, Dinnbier U, Epstein W, Gajewska A (1987). Evidence for multiple K^+^ export systems in *Escherichia coli*. J. Bacteriol..

[50] Shannon RD (1976). Revised effective ionic radii and systematic studies of interatomic distances in halides and chalcogenides. Acta Cryst. A.

[51] Yao Z, Weinberg Z, Ruzzo WL (2006). CMfinder—a covariance model based RNA motif finding algorithm. Bioinformatics.

[52] Nawrocki EP, Eddy SR (2013). Infernal 1.1: 100-fold faster RNA homology searches. Bioinformatics.

[53] Weinberg Z, Breaker RR (2011). R2R-software to speed the depiction of aesthetic consensus RNA secondary structures. BMC Bioinform..

[54] Rivas E, Clements J, Eddy SR (2017). A statistical test for conserved RNA structure shows lack of evidence for structure in lncRNAs. Nat. Methods.

[55] Altschul SF, Gish W, Miller W, Myers EW, Lipman DJ (1990). Basic local alignment search tool. J. Mol. Biol..

[56] Li Y, Breaker RR (1999). Kinetics of RNA degradation by specific base catalysis of transesterification involving the 2′-hydroxyl group. J. Am. Chem. Soc..

[57] Landick R, Wang D, Chan CL (1996). Quantitative analysis of transcriptional pausing by *Escherichia coli* RNA polymerase *his* leader pause site as paradigm. Meth. Enzymol..

[58] Sudarsan N, Wickiser JK, Nakamura S, Ebert MS, Breaker RR (2003). An mRNA structure in bacteria that controls gene expression by binding lysine. Genes Dev..

[59] Atilho, R. M., Arachchilage, G., Greenlee, E. B., Knecht, K. M. & Breaker, R. R. A bacterial riboswitch class for the thiamin precursor HMP-PP employs a terminator-embedded aptamer. *eLife* **8**, e45210 (2019). 10.7554/eLife.45210 PMC6478431 30950790

[60] Stancik LM (2002). pH-dependent expression of periplasmic proteins and amino acid catabolism in *Escherichia coli*. J. Bacteriol..

