# Supplementary material for: Na+ riboswitches regulate genes for diverse physiological processes in bacteria
Source: Nat Chem Biol. 2022 Jul 25;18(8):878–85. doi: 10.1038/s41589-022-01086-4 (PMC9337991; doi:10.1038/s41589-022-01086-4)
Supplement: Supplementary file 1 — Supplemental Tables 1–3. [file 41589_2022_1086_MOESM1_ESM.pdf]

nature portfolio

https://doi.org/10.1038/s41589-022-01086-4

Supplementary information

Na^+ riboswitches regulate genes for diverse physiological processes in bacteria

In the format provided by the authors and unedited

Supplementary Information

Na^+ riboswitches regulate genes for diverse physiological processes in bacteria

Neil White[1,2,4], Harini Sadeeshkumar[1,4], Anna Sun[1], Narasimhan Sudarsan[2], and Ronald R. Breaker[1,2,3,5]

[1]Department of Molecular, Cellular and Developmental Biology, Yale University, New Haven, CT 06520-8103, USA;

[2]Howard Hughes Medical Institute, Yale University, New Haven, CT 06520-8103, USA;

[3]Department of Molecular Biophysics and Biochemistry, Yale University, New Haven, CT 06520-8103, USA

[4]These authors contributed equally to this work.

[5]To whom correspondence may be addressed. e-mail: ronald.breaker@yale.edu

Supplementary Table 1. Quantitation of riboswitch-reporter fusion assays. *B. subtilis* cells carrying the WT or M4 riboswitch-reporter constructs depicted in **Fig. 3a** were grown overnight in low-sodium (~15 mM Na^+) LB (yeast extract and tryptone). 20 µL of the overnight culture was subcultured in low-sodium LB buffered at pH 7.0 (100 mM PIPES), pH 8.0 (100 mM TAPS), or pH 9.0 (100 mM AMPSO), all containing chloramphenicol (5 µg/mL). As indicated for each assay, ions were added to supplement the media, the mixtures were incubated overnight, and ONPG (ortho-nitrophenyl-ß-galactoside) was added to samples to measure β-galactosidase activity (units presented in the table) by adapting the method described previously [Miller, J.H. Procedures for working with lac. In: A Short Course in Bacterial Genetics. (Cold Spring Harbor, NY: Cold Spring Harbor Laboratory Press) pp 72 (1992)]. Three independent replicates of bacterial cultures and gene expression assays were performed for each condition. The values are plotted in Extended Data Fig. 5.

pH	Construct	Conditions	1	2	3	Mean	SD
pH 7	WT	no ligand	93.41	94.30	76.02	87.91	10.30
	WT	300 mM Na^+	146.77	160.13	131.85	146.25	14.15
pH 8	WT	no ligand	54.07	49.01	43.98	49.02	5.05
	WT	300 mM Na^+	166.89	110.43	103.17	126.83	34.88
pH 9	WT	no ligand	45.43	40.08	51.45	45.65	5.69
	WT	300 mM Na+	191.89	191.49	225.88	203.09	19.74
	M4	no ligand	45.23	39.03	64.75	49.67	13.43
	M4	300 mM Na+	44.94	43.09	63.56	50.53	11.32
	WT	75 mM Li^+	129.95	79.59	83.19	97.58	28.09
	WT	300 mM K+	60.35	78.26	90.18	76.26	15.02
	WT	30 mM Mg^{2+}	65.57	74.46	77.91	72.65	6.36
	WT	1 mM Ca^{2+}	38.57	39.34	44.07	40.66	2.98
	WT	1 mM Sr^{2+}	50.61	50.54	42.44	47.87	4.70
	WT	1 mM Ba^{2+}	91.72	51.57	73.47	72.25	20.11

Supplementary Table 2. Examples of tandem Na^+ and c-di-AMP riboswitches. The metabolic products of the enzymes encoded by the associated genes have been implicated as osmolytes in bacteria. The construct depicted in **Fig. 4A** is from *Dehalobacter sp. CF*.

Assession Number	Organism	Annotated Downstream Gene	Description
NZ_JQKC01000001.1/393024-392971	*Pseudobacteroides cellulosolvens*	*dapB*	Dihydrodipicolinate reductase (4-hydroxy-tetrahydrodipicolinate reductase)
NC_018867.1/92466-92413	*Dehalobacter sp. CF*	*ablA*	L-Lysine-2,3-aminomutase
NZ_CP007033.1/962972-962918	*Dehalobacter restrictus*	*ablA*	L-Lysine-2,3-aminomutase
JGI1356J14229_10144053/201-147	Species unknown: metagenomic	No data	
NZ_JH556658.1/548863-548918	*Acetivibrio cellulolyticus*	*dapB*	Dihydrodipicolinate reductase (4-hydroxy-tetrahydrodipicolinate reductase)
NC_013216.1/623238-62330	*Desulfotomaculum acetoxidans*	*eam*	Glutamate 2,3-aminomutase
NC_009454.1/609797-609856	*Pelotomaculum thermopropionicum*	*eam*	Glutamate 2,3-aminomutase

Supplementary Table 3. Synthetic DNAs. Red nucleotides designate the T7 RNA polymerase promoter.

In-line Probing Constructs

C. acetobutylicum 66 *kefB* (WT) DNA Template:
TAATACGACTCACTATAGGATTTCGGTTGAGCAAATTTATATTTGTTTTCAAGAGCTTTGAAT
TTCTGGGGTGGGAAATTCCTTA

Forward primer:
TAATACGACTCACTATAGGATTTCGGTTGAGCAAATTTATATTTGTTTTCAAGA

Reverse primer:
TAAGGAATTTCCCACCCCAGAAATTCAAAGCTCTTGAAAACAAATATAAATTTG

(M1):
Forward Primer:
TAATACGACTCACTATAGGATTTCGGTTGAGCAAATTTATATTTGTTTTCTTGA

Reverse Primer:
TAAGGAATTTCCCACCCCAGAAATTCAAAGCTCAAGAAAACAAATATAAATTTG

(M2):
Forward primer: Same as for WT

Reverse Primer:
TAAGGAATTTCCCACAACAGAAATTCAAAGCTCTTGAAAACAAATATAAATTTG

L. garvieae Lg2 53 *mgtA:*
TAATACGACTCACTATAGGATATAGGTTGGGCGCAAGCTTCAAGAGATTTCCTCCGAGGGTA
AGGAGGAGAT

Forward Primer:
TAATACGACTCACTATAGGATATAGGTTGGGCGCAAGCTTCAAGAGATTT

Reverse Primer:
ATCTCCTCCTTACCCTCGGAGGAAATCTCTTGAAGCTTGCGCCCAACCTA

T. thermosaccharolyticum M0795 66 DUF1646:
TAATACGACTCACTATAGGTTTGCGGTTGAACAGGCTTTATAGCTTGCTTCAAGATTTCTGGC
TTCCAGGGTGGGAAGCTCATCA

Forward Primer:
TAATACGACTCACTATAGGTTTGCGGTTGAACAGGCTTTATAGCTTGCTTCAAG

Reverse Primer:
TGATGAGCTTCCCACCCTGGAAGCCAGAAATCTTGAAGCAAGCTATAAAGCCTG

DUF1646 Transcription Termination Constructs

DUF1646_WT
GCCGTACGACGAATTCGAATTAAATATTGATATACATCAATTAATCTGTTATGATTATATTAA
ATAAAATATTTTAACATTTCGGTTGAGCAAATTTATATTTGTTTTCAAGAGCTTTGAATTTCT
GGGGTGGGAAATTCCTTAAAAGAGGAATATCCTACCTTTTTTGCGTACAAAATATTATTTGG
AGGTGTGATTTTTGGACAAGATCTTATTTGATATTGGATCCAAAGGA

DUF1646_M3
GCCGTACGACGAATTCGAATTAAATATTGATATACATCAATTAATCTGTTATGATTATATTAA
ATAAAATATTTTAACATTTCGGAAGAGCAAATTTATATTTGTTTTCAAGAGCTTTGAATTTCT
GGGGTGGGAAATTCCTTAAAAGAGGAATATCCTACCTTTTTTGCGTACAAAATATTATTTGG
AGGTGTGATTTTTGGACAAGATCTTATTTGATATTGGATCCAAAGGA

DUF1646_M4
GCCGTACGACGAATTCGAATTAAATATTGATATACATCAATTAATCTGTTATGATTATATTAA
ATAAAATATTTTAACATTTCGGTTGAGCAAATTTATATTTGTTTTCAAGAGCTTTGAATTTCT
GTGGTGGGAAATTCCTTAAAAGAGGAATATCCTACCTTTTTTGCGTACAAAATATTATTTGG
AGGTGTGATTTTTGGACAAGATCTTATTTGATATTGGATCCAAAGGA

DUF1646_antiterm
GCCGTACGACGAATTCGAATTAAATATTGATATACATCAATTAATCTGTTATGATTATATTAA
ATAAAATATTTTAACATTTCGGTTGAGCAAATTTATATTTGTTTTCAAGAGCTTTGAATTTCT
GGGGTGGGAAATTCCTTAAAAGAGGAATATCCTACCAAAAAAGCGTACAAAATATTATTTG
GAGGTGTGATTTTTGGACAAGATCTTATTTGATATTGGATCCAAAGGA

DUF1646_FP
GCCGTACGACGAATTCGAATTAAATATTGA

DUF1646_RP1
TCCTTTGGATCCAATATCAAATAAGATCTT

DUF1646_cdA_WT
TACGACGAATTCTACGCTAGACTTGATCCTTTTAAATAAGTCTGATAAAATGTGAACTAATTT
ATAAGGTTACTTTCGGTTGGAATGGTTATTACCATTTTCAAGAGAAGCGGGCTCTGGGGTGG
GAGGCTACCAAATATTGGTATAGCTATCCACCTTTATTTATTTTTTTTAAGACAACAACTGAA
TATATTTATACGCTGAATCCGTTAGGAACGGGAGACCCAAATAATAGGGGTGAATCCAAAG
CGGTTGCTTTGGTAGGGTTAAGCTCTTTCGACCCGAATCCGTCAGCTAATCTCGTAAGCGGT
GGAAGAGAAGGTGATTGATATGCTTAATTACACCTTTAGTTAAGGTATATATTTTAGCATAC
GCTGCAGATACTTCTCTGCAGCTTTTTGTTTTATATACCACAAATGTTGAGGATGACAATCTT
CAATGAGAATCTGAAGGAGGAATTAAATAATATGGGATCCAAAGGA

DUF1646_cdA_antiterm
TACGACGAATTCTACGCTAGACTTGATCCTTTTAAATAAGTCTGATAAAATGTGAACTAATTT
ATAAGGTTACTTTCGGTTGGAATGGTTATTACCATTTTCAAGAGAAGCGGGCTCTGGGGTGG
GAGGCTACCAAATATTGGTATAGCTATCCACCAAAAAAAATTTTTTTTAAGACAACAACTGA
ATATATTTATACGCTGAATCCGTTAGGAACGGGAGACCCAAATAATAGGGGTGAATCCAAA
GCGGTTGCTTTGGTAGGGTTAAGCTCTTTCGACCCGAATCCGTCAGCTAATCTCGTAAGCGG
TGGAAGAGAAGGTGATTGATATGCTTAATTACACCTTTAGTTAAGGTATATATTTTAGCATA
CGCTGCAGATACTTCTCTGCAGCAAAAAGAAAAATATACCACAAATGTTGAGGATGACAAT
CTTCAATGAGAATCTGAAGGAGGAATTAAATAATATGGGATCCAAAGGA

DUF1646_cdA_FP
TACGACGAATTCTACGCTAGACTTGATCCT

DUF1646_cdA_RP
TCCTTTGGATCCCATATTATTTAATTCCTC

Genetics Constructs

DUF1646_WT
GCCGTACGACGAATTCGAATTAAATATTGATATACATCAATTAATCTGTTATGATTATATTAA
ATAAAATATTTTAACATTTCGGTTGAGCAAATTTATATTTGTTTTCAAGAGCTTTGAATTTCT
GGGGTGGGAAATTCCTTAAAAGAGGAATATCCTACCTTTTTTGCGTACAGGATCCAAAGGA

(Also used DUF1646_FP)
NW_Duf1646_RP2
TCCTTTGGATCCTGTACGCAAAAAAGGTAG
