# Supplementary figures and images for: Na+ riboswitches regulate genes for diverse physiological processes in bacteria
Source: Nat Chem Biol. 2022 Jul 25;18(8):878–85. doi: 10.1038/s41589-022-01086-4 (PMC9337991; doi:10.1038/s41589-022-01086-4)

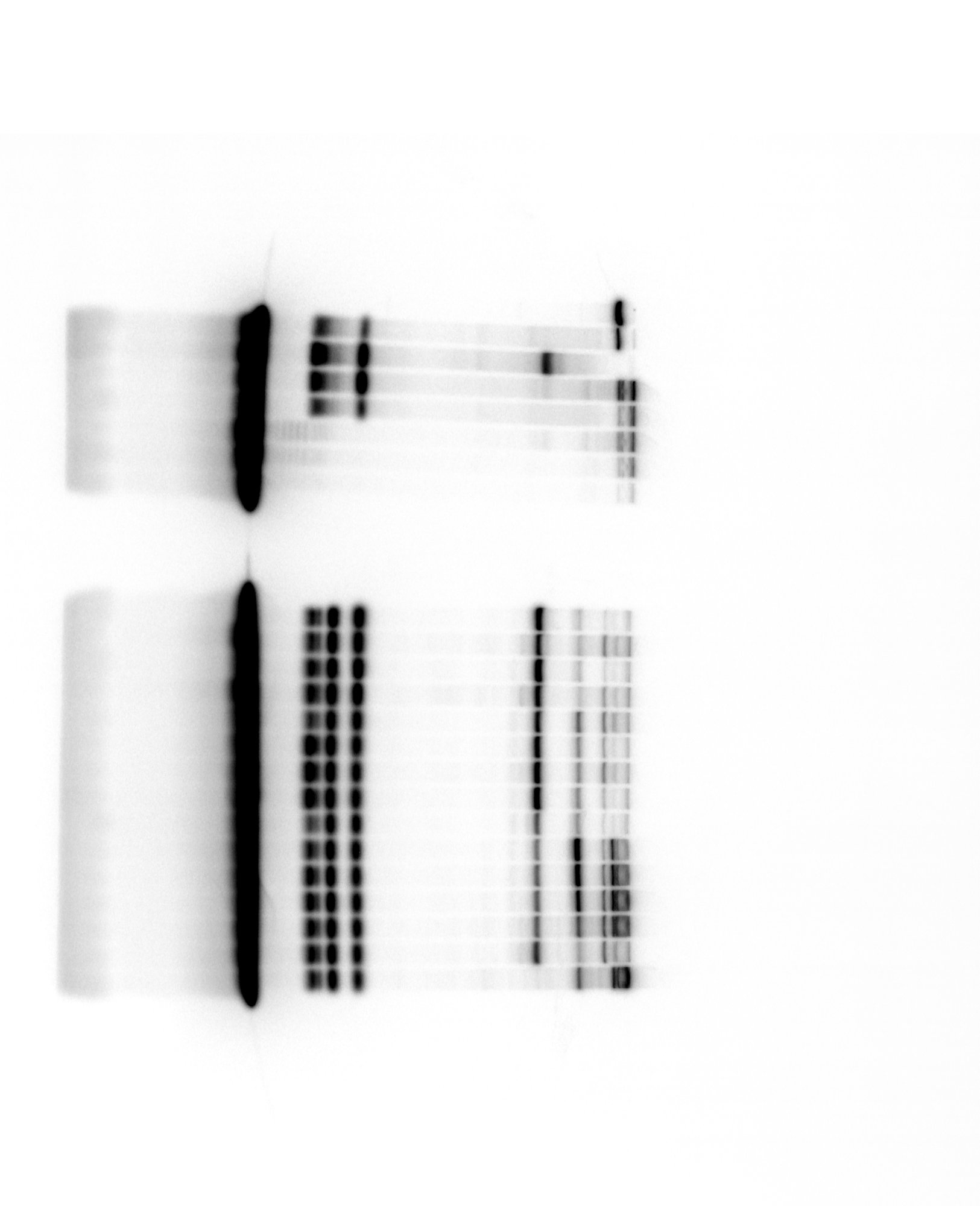

Supplement: Source Data Fig. 3 — Uncropped gel from Fig. 3b. [file 41589_2022_1086_MOESM3_ESM.jpg]

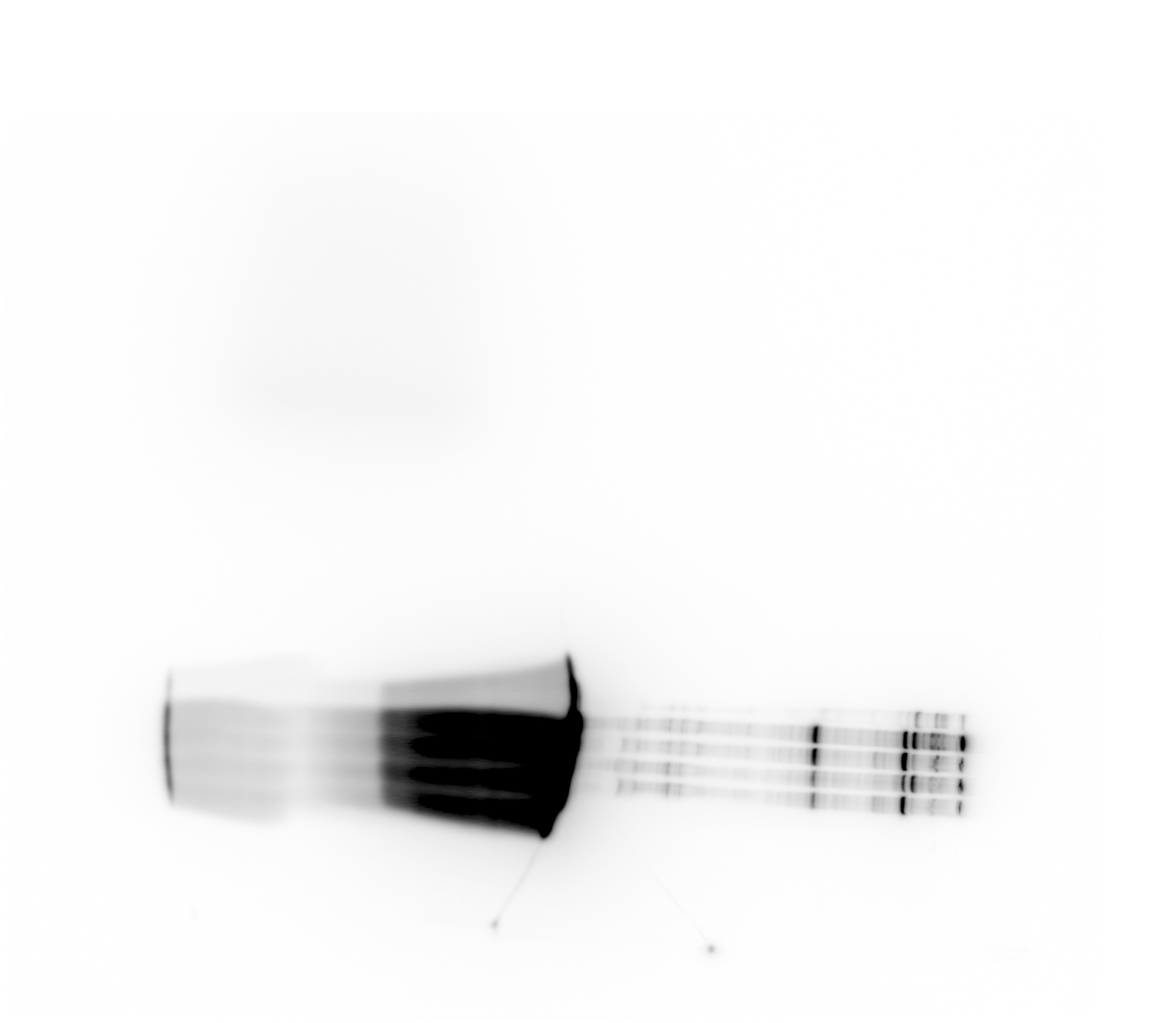

Supplement: Source Data Fig. 4 — Uncropped gel from Fig. 4c. [file 41589_2022_1086_MOESM4_ESM.jpg]
